# Prediction early recurrence of hepatocellular carcinoma eligible for curative ablation using a Radiomics nomogram

**DOI:** 10.1186/s40644-019-0207-7

**Published:** 2019-04-26

**Authors:** Chunwang Yuan, Zhenchang Wang, Dongsheng Gu, Jie Tian, Peng Zhao, Jingwei Wei, Xiaozhen Yang, Xiaohan Hao, Di Dong, Ning He, Yu Sun, Wenfeng Gao, Jiliang Feng

**Affiliations:** 1 Department of Radiology, Beijing Friendship Hospital, Capital Medical University, No.95, Yong An Road, Xicheng District, Beijing, 100050 China; 2 Center of Interventional Oncology and Liver Diseases, Beijing Youan Hospital, Capital Medical University, Beijing, 100069 China; 3 Key Laboratory of Molecular Imaging, Institute of Automation, Chinese Academy of Sciences, No.95 Zhongguancun East Road, Haidian District, Beijing, 100190 China; 4 Center of Clinical Pathology, Beijing Youan Hospital, Capital Medical University, Beijing, 100069 China; 5 Beijing Advanced Innovation Center for Big Data-Based Precision Medicine, School of Medicine, Beihang University, Beijing, 100191 China; 6 Engineering Research Center of Molecular and Neuro Imaging of Ministry of Education, School of Life Science and Technology, Xidian University, Xi’an, Shanxi 710126 China; 7 University of Chinese Academy of Sciences, Beijing, 100049 China

**Keywords:** Hepatocellular carcinoma, Radiomics, Recurrence, forecasting, Ablation techniques

## Abstract

**Background:**

Predicting early recurrence (ER) after radical therapy for HCC patients is critical for the decision of subsequent follow-up and treatment. Radiomic features derived from the medical imaging show great potential to predict prognosis. Here we aim to develop and validate a radiomics nomogram that could predict ER after curative ablation.

**Methods:**

Total 184 HCC patients treated from August 2007 to August 2014 were included in the study and were divided into the training (*n* = 129) and validation(*n* = 55) cohorts randomly. The endpoint was recurrence free survival (RFS). A set of 647 radiomics features were extracted from the 3 phases contrast enhanced computed tomography (CECT) images. The minimum redundancy maximum relevance algorithm (MRMRA) was used for feature selection. The least absolute shrinkage and selection operator (LASSO) Cox regression model was used to build a radiomics signature. Recurrence prediction models were built using clinicopathological factors and radiomics signature, and a prognostic nomogram was developed and validated by calibration.

**Results:**

Among the four radiomics models, the portal venous phase model obtained the best performance in the validation subgroup (C-index = 0.736 (95%CI:0.726–0.856)). When adding the clinicopathological factors to the models, the portal venous phase combined model also yielded the best predictive performance for training (C-index = 0.792(95%CI:0.727–0.857) and validation (C-index = 0.755(95%CI:0.651–0.860) subgroup. The combined model indicated a more distinct improvement of predictive power than the simple clinical model (ANOVA, *P* < 0.0001).

**Conclusions:**

This study successfully built a radiomics nomogram that integrated clinicopathological and radiomics features, which can be potentially used to predict ER after curative ablation for HCC patients.

**Electronic supplementary material:**

The online version of this article (10.1186/s40644-019-0207-7) contains supplementary material, which is available to authorized users.

## Background

Hepatocellular carcinoma (HCC) is the fifth most common cancer and the third leading cause of cancer-related death globally [1], In China, it ranks fourth and second respectively [2]. Surgical resection and liver transplantation are standard treatments for early HCC, but their applications are limited because the severe liver dysfunction, concomitant diseases and the shortage of liver grafts. Therefore, several non-surgical local ablation techniques have been introduced, such as radiofrequency ablation (RFA), microwave ablation (MWA), and cryoablation (Cryo-A) [3]. These methods are currently widely used because they are simple, safe, effective, minimally invasive, repeatable, and require a short hospital stay. Hence, they are often considered the best options for HCC patients with Barcelona Clinic Liver Cancer (BCLC) stage 0-A and some selected BCLC stage B (Combined with transcatheter arterial chemoembolization (TACE)) [4–7], who are not suitable for resection or liver transplantation.

ER after ablation (Intrahepatic new lesion occurs within 2 years after ablation) is one of the main factors of mortality [8–12].Patients with late recurrence (Intrahepatic new lesion occurs after 2 years post-ablation) could have a better survival than patients with ER [11, 12]. Therefore, early detection and timely treatment of HCC recurrence should improve prognosis [13], but up to now there is no really powerful tool for predicting ER after ablation.

A number of prognostic scoring systems and nomograms were developed to predict the risk of HCC recurrence after radical resection [14–16]. These systems are based on demographic, clinical, and biochemical factors that may be associated with tumor recurrence. CECT imaging can provide information and enhancement features of the entire tumor, and has been reportedly used to achieve 78% reconstruction of the global HCC gene-expression profiles by the combination of 28 morphologic imaging traits [17], thus making it possible to infer the biological behavior of the tumor through the imaging features [18–20]. Radiomics extracts large amounts of quantitative features from medical images to reveal disease characteristics that fail to be detected by the naked eye [18, 19, 21–24]. Radiomics features specific to each patient provide valuable information for personalized medicine [23]. In this light, radiomics could improve the prediction of HCC ER. A recent study showed that radiomics signatures were predictive for HCC ER after surgical resection [25].We want to develop and validate radiomics signatures that could predict RFS after curative ablation in order to actively adopt tailored follow-up strategies and interventions.

## Methods

### Patients and laboratory, pathology, imaging data

**Fig. 1:**
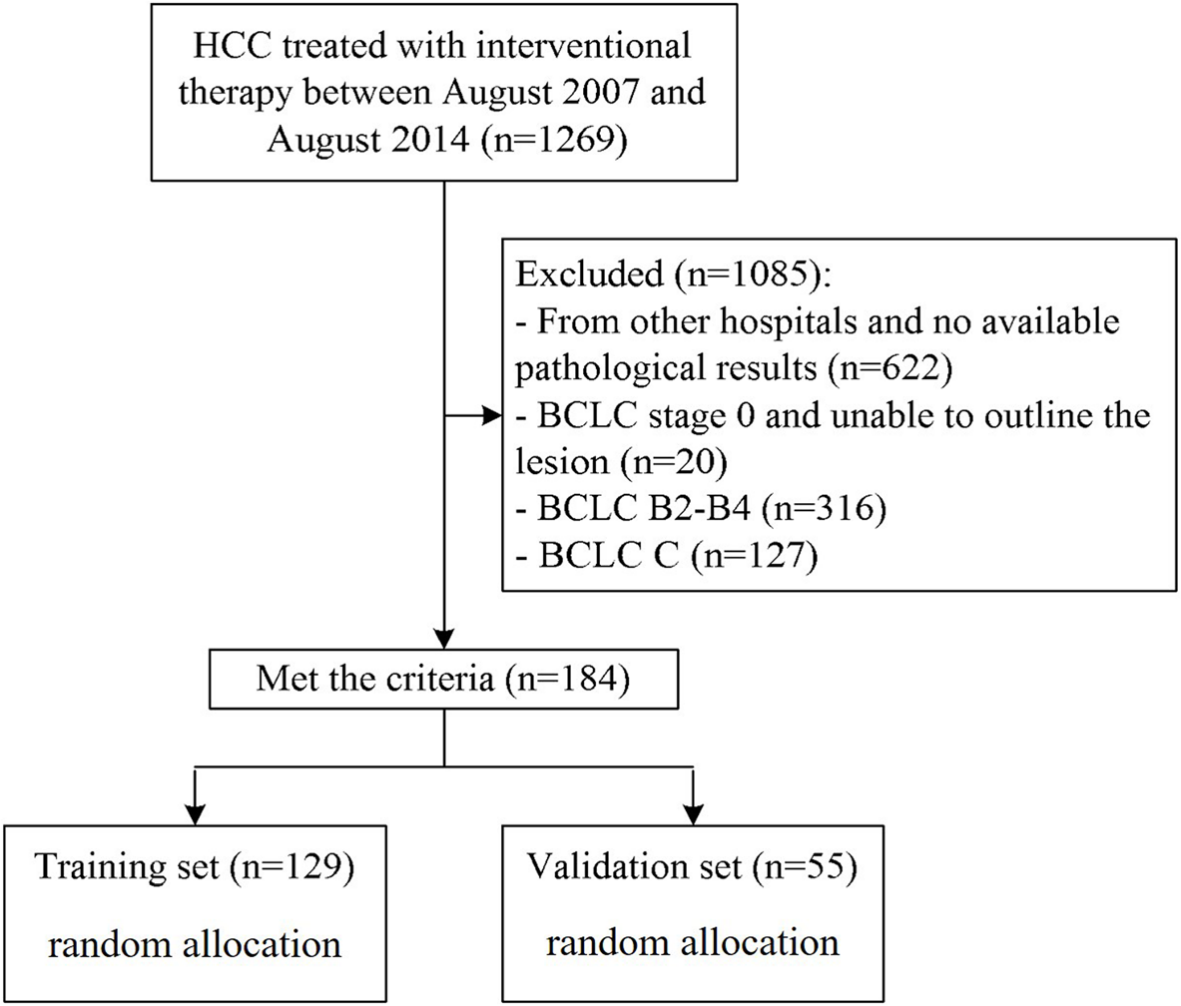
Patient flowchart

The retrospective study was approved by institution ethics committee, which enrolled 184 HCC patients hospitalized from August 2007 to August 2014 and gained curative ablation. All ablation procedures were performed by CT-guided percutaneous route, which were performed by Interventional radiologists with more than 500 ablation procedures experience. Patients were randomly divided into training cohort (*n* = 129) and validation cohort (*n* = 55). The human experimentation guidelines of the PRC were followed, the informed consent was not required. All patients were diagnosed with HCC by percutaneous biopsy pretherapy. The CECT images were entirely from a 64-row spiral CT system (Lightspeed VCT, GE Healthcare, Pittsburgh, PA, USA), with format of digital imaging and communications in medicine (DICOM). The pathological data were obtained totally from of hospital pathology center. Figure 1 shows the patient flowchart. The inclusion criteria: 1) 18–75 years of age; 2) CECT was performed within 2 weeks before ablation; 3) BCLC stage A to B2; 4) unwilling to undergo hepatectomy or liver transplantation; 5) well-preserved liver function, i.e., Child-Pugh class A/B, serum total bilirubin level ≤ 3 mg/dl; and 6) eastern cooperative oncology group (ECOG) performance status score ≤ 2. The exclusion criteria: 1) tumor thrombus in a major hepatic vessel; 2) extrahepatic metastases; 3) uncontrollable ascites, history of hepatic encephalopathy, or variceal bleeding occurred less than 1 month; 4) Child-Pugh class C; 5) severe coagulation disorder (platelet count less than 5 × 10^3^/μLor prothrombin activity <50%; 6) history of secondary malignancy; 7) severe dysfunction of the heart, brain, kidney, or other organs; 8) active infection (except viral hepatitis); or 9) refusal of ablation. Follow-up tactics: All patients underwent liver CECT/CEMRI scan, test of liver function and tumor markers (such as AFP) every 3 months after ablation. The shortest follow-up time was>3years. Tumor recurrence was diagnosed by CECT/CEMRI showing arterial hyperenhancement and wash out appearance in the portal venous phase [26].The baseline characteristics of enrolled patients were listed in Table 1.

**Table 1:** Patient characteristics in the training and validation datasets

Characteristics	Training dataset (*N* = 129)	Validation dataset (*N* = 55)	P
Age^a^, median (IQR), years	57 (49–63)	57 (51–61)	0.652
Gender, n (%)			0.673
Male	95 (73.6)	41 (74.5)	
Female	34 (26.4)	14 (25.5)	
Tumor diameter^a^, median (IQR), cm	3.3 (2.5–4.7)	3.5 (2.7–5.2)	0.384
Histological grade, n (%)			0.086
I	31 (24.0)	16 (29.1)	
II	66 (51.2)	32 (58.2)	
III	32 (24.8)	7 (12.7)	
AFP^a^, median (IQR)	14.2 (3.6–144.3)	20.5 (4.9–132.3)	0.128
Cause, n (%)			0.467
Alcohol	7 (5.4)	4 (7.3)	
HCV	14 (10.9)	6 (10.9)	
HBV	106 (82.2)	45 (81.8)	
HBV + HCV	2 (1.6)	0	
ECOG, n (%)			0.614
0	3 (2.3)	0	
1	126 (97.7)	55 (100)	
CK19+, n (%)			0.633
0	107 (82.9)	46 (83.6)	
1	17 (13.2)	7 (12.7)	
2	1 (0.8)	1 (1.8)	
3	4 (3.1)	1 (1.8)	
GPC3+, n (%)			0.833
0	40 (31)	13 (23.6)	
1	47 (36.4)	22 (40)	
3	25 (19.4)	9 (16.4)	
4	17 (13.2)	11 (20)	
HBsAg+, n (%)			0.960
0	74 (57.4)	33 (60)	
1	38 (29.5)	20 (36.4)	
2	12 (9.3)	2 (3.6)	
3	5 (3.9)	0	
HBcAg+, n (%)			0.328
0	121 (93.8)	48 (87.3)	
1	7 (5.4)	7 (12.7)	
2	1 (0.8)	0	
Child-Pugh, n (%)			0.102
0	122 (94.6)	55 (100)	
1	7 (5.4)	0	
BCLC, n (%)			0.382
A	81 (62.8)	27 (49.1)	
B1	48 (37.2)	28 (50.9)	
Ablation approach			0.175
RFA	86 (66.7)	39 (70.9)	
MWA	27 (20.9)	14 (25.5)	
CRYO-A	16 (12.4)	2 (3.6)	
RFS time^a^, median (IQR), months	15 (6–30)	13 (5–36)	0.799

*Note:* Data are shown as number of patients, with the percentage in parentheses unless noted. No significant differences were found between the training cohort and the validation datasets

^a^Data are medians, with interquartile ranges in parentheses

### Statistical analysis

Individual variables were analyzed for significant differences in the training and validate cohort using the Mann Whitney U test for continuous variables and x^2^ test for categorical variables. All statistical analyses were implemented using PASW Statistics 18.0.0 and R (version 3.4.1). *P*-values (Two-sided) < 0.05 were regarded as statistically significant.

### Tumor segmentation, radiomics feature extraction, Clinicopathological factors analysis, clinical model building, Radiomics feature selection, Radiomics signature building and model evaluation

#### Tumor segmentation

Three-dimensional manual segmentation of tumors were performed by a radiologist with more than 15 yrs. work experience, using the ITK-SNAP software (http://www.itksnap.org/pmwiki/pmwiki.php). Regions of interest (ROI) were drawn on the images from the arterial, portal venous, and parenchymal phases, slice by slice, for each patient [25, 27]. The final segmentation results were validated by a senior radiologist with more than 20 yrs. work experience to ensure segmentation validity. Test-retest datasets were obtained to test the reproducibility of the extracted features in 20 patients randomly selected for repeated segmentation by the senior radiologist.

#### Radiomics feature extraction

A set of 647 radiomics features that reflected the machine-read radiological characteristics and subtle textural information was extracted from the segmented ROIs. Image filtering was implemented on original 3-dimension tumor slices with undecimated wavelet transform [27], which decomposed the original image into eight decompositions. Features were extracted from both the original and filtered images and could be divided into two types: non-textural features and textural features. The non-textural features included shape, size, and intensity features. Shape and size features captured the direct-viewing characteristics of the lesion. Intensity features depicted the characteristics of the histogram of the tumor lesion. Textural features were extracted based on four textural matrixes: Gray Level Co-occurrence Matrix (GLCM), Gray Level Run-Length Matrix (GLRLM), Gray Level Size Zone Matrix (GLSZM), and Neighborhood Gray-Tone Difference Matrix (NGTDM) [28–30]. All features’ types and names are presented in Additional file 1: Table S2. Features extraction was performed using Matlab 2014a (MathWorks, Natick, MA, USA).

#### Clinicopathological factors and clinical model building

Clinicopathological factors with *P*-values < 0.10 in univariable Cox proportional hazard regression analysis were integrated into a stepwise multivariable Cox model. Variables with P-values < 0.05 in the multivariable analysis were identified as potential clinicopathological factors related to RFS and were included for the clinical model building.

#### Radiomics feature selection

The intra-class correlation coefficient (ICC) was calculated to determine the stability of the features, followed by the test-retest setting. Features with an ICC < 0.75 were excluded from the final feature set. In order to reduce the redundancy and the unnecessary complexity for computation and modeling, MRMRA was used for feature selection. The aim of this algorithm was to select a feature subset which can achieve the best characterization of the difference between the two targeted classified groups, considering the restriction that these features were mutually dissimilar with each other to the full extent, but marginally related to the selected clinical outcome [31]. Moreover, MRMRA was proved to be more stable for the feature extraction process, especially for radiomics [32]. A set of 20 potential features were selected for further model construction according to the output score of each feature by MRMRA. The “irr” R package was used for computation of intra-class correlation coefficient. The “mRMRe” R package was used for MRMRA feature selection.

#### Radiomics signature building

We used the extensive LASSO method for further variable selection in Cox proportional hazards model with the 20 selected features from the training dataset [33, 34]. We chose the optimal feature set that had the maximal cross-validation log partial likelihood. Non-zero coefficients were defined as the weight for each selected feature, which indicated the hazard ratio between the feature and survival. Each patient’s radiomics signature was generated by multiplying the selected features with their respective coefficients. We utilized the LASSO Cox regression to build prediction models on the training dataset and validated their predictive effectiveness using the validation cohort. The prognostic value of the radiomics signature was estimated by Kaplan-Meier (KM) curves. Patients were stratified into the high and low risk groups through the median value of radiomics signature (arterial phase: 3.037; portal venous phase: 2.687, and parenchymal phase: 2.493). We compared the two KM survival curves by the log-rank test. The glmnet R package was used for LASSO Cox variable selection and model building. The survival R package was used for comparison of the survival curves.

#### Model evaluation

The final Cox proportional hazard model incorporated the clinicopathological factors along with three phases radiomics signatures. The performance of the clinical model, radiomics models, and the final combined model were evaluated by the concordance index (C-Index) [27] with 1000 bootstrap resamples, which is the area under the curve for continuous time-to-event survival data and can measure the discrimination of a prognostic model by the area under the curve of continuous time-to-event survival data. A value of 1 indicates perfect discrimination and 0.5 represents discriminative power equal to randomness. The Hosmer-Leme show test was applied for the prediction model [35]. We further built a nomogram for the model to provide a more direct way to determine the 1-, 2- and 3-year RFS rates. A calibration curve was plotted to analyze the prognostic performance of the nomogram on both the training and validation datasets [36]. The “rms” R package was used for Cox proportional hazards regression, nomograms, and calibration curves.

## Results

1. Demographic data are provided in Table 1.
2. The results of uni- and multivariable analyses for the clinical model construction are showed in Table 2.
3. Radiomics feature selection and model construction.

**Table 2:** Results of the univariable and multivariable analyses

Clinical predictors	Univariable		Multivariable	
	P	HR (95%CI)	P	HR (95%CI)
Gender	0.57	1.159 (0.697–1.927)		
Age	0.527	1.004 (0.992–1.016)		
Tumor maximal diameter	0.59	0.975 (0.888–1.07)		
Grade	0.099	0.791 (0.599–1.045)	0.3442	0.889 (0.696–1.135)
AFP	0.932	1 (1–1)		
Etiology	0.28	0.823 (0.577–1.172)		
ECOG	0.129	4.798 (0.634–36.289)		
CK19+	0.46	1.127 (0.821–1.545)		
GPC3+	0.251	1.134 (0.915–1.406)		
HBsAg+	0.714	1.054 (0.797–1.393)		
HBcAg+	0.713	0.856 (0.375–1.955)		
Child-Pugh	0.087	2.128 (0.897–5.051)	0.007*	2.762 (1.317–5.791)
BCLC	<0.001	2.145 (1.55–2.967)	<0.001*	4.834 (2.698–8.662)

Note: * *P* < 0.05

**Fig. 2:**
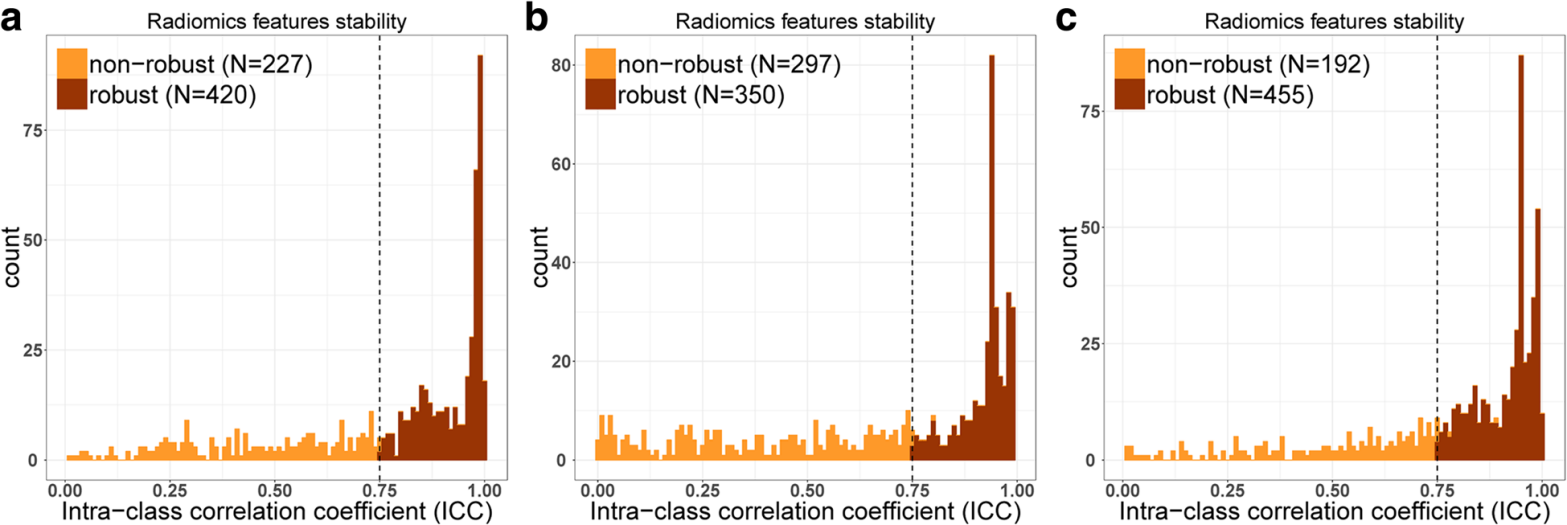
Histogram of the intra-class correlation coefficient (ICC). For the 20 random selected patients from the overall dataset, we extracted the radiomics features from the test and re-test scans. The ICC was used to determine the stability of the features. Features with an ICC <0.75 were excluded from the analysis. After robustness test, 420 of the initial 647 CT image features in the arterial phase, 350 in the portal venous phase, and 455 in the parenchymal phase were retained

Presented in Fig. 2. The top 20 features were adopted as inputs to the LASSO Cox model [34, 35]. According to the leave-one-out cross validation, the final numbers of features included were 5, 5, and 10 for the radiomics model construction in the arterial, portal venous, and parenchymal phases, respectively. The selected features are shown in supporting information (Additional file 1: Table S1), and the detailed information is presented in supporting information (Additional file 1: Table S2). The corresponding formulas of the radiomics signature for the three phases are separately shown in supporting information (Additional file 1: Table S3). Univariate Cox proportional hazard model was built with a single radiomics signature from the three phases CECT images respectively, and multivariable Cox proportional hazard model integrated the three phases radiomics signatures was built as a fusion model.

4. Validation of the radiomics signature

**Fig. 3:**
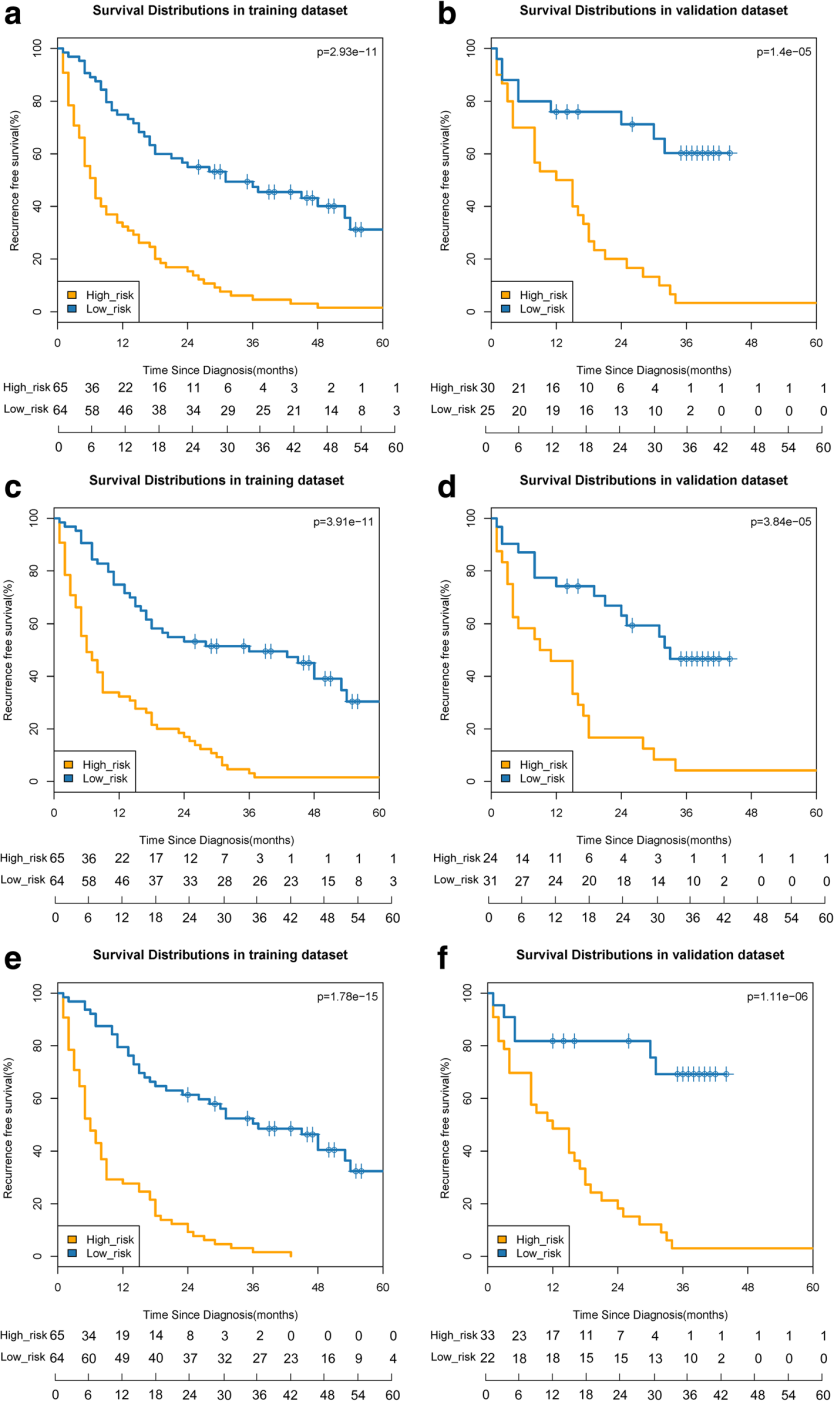
Kaplan-Meier analyses of recurrence-free survival based on the proposed signature with cut-off values as the median of the training dataset. **a** Training dataset in the arterial phase. **b** Validation dataset in the arterial phase. **c** Training dataset in the portal venous phase. **d** Validation dataset in the portal venous phase. **e** Training dataset in the parenchymal phase. **f** Validation dataset in the parenchymal phase

Each single radiomics signature satisfied the discriminative power in the univariable Cox model. The arterial, portal venous, parenchymal phase signatures yielded HR of 11.46(*P* < 0.0001, 95%CI: 4.14–31.68), 20.00(*P* = 0.0002, 95%CI: 4.14–96.61), 6.16 (P < 0.0001, 95%CI: 3.35–11.34), respectively. KM curves for all phases on both the training and validation datasets were developed (Fig. 3). The log-rank test revealed a significant difference (*P* < 0.001) between the high- and low-risk subgroups for all phases.

5. Performance of the different models

A validation dataset with 55 patients included randomly was used to evaluate the models’ predictive power. One clinical model, four radiomics models, and four combined models were built. The clinical model had the worst predictive performance in the training dataset with a C-index of 0.649 (95%CI: 0.592–0.706) and the validation dataset with a C-index of 0.556 (95%CI: 0.471–0.641). Among the four radiomics models, the fusion model with three phases radiomics signatures had the best predictive performance for RFS, with a C-index of 0.791 (95%CI: 0.726–0.856) in the training dataset, but did not show the best performance in the validation dataset, with a C-index of 0.690 (95%CI: 0.586–0.795). The portal venous phase radiomics model obtained the best performance in the validation dataset with a C-index of 0.736 (95%CI: 0.726–0.856). When adding the clinicopathological factors to the four radiomics models, the combined model consisting of the portal venous phase radiomics signatures yielded the best predictive power in the validation dataset (C-index = 0.755 [95%CI: 0.651–0.860]), which also indicated a distinct improvement for the clinical model with a significant difference (ANOVA, *P* < 0.0001) (Table 3). RFS during follow-up and the 1-, 2- and 3-year recurrence rates in both subgroups of portal venous phase are listed in Table 4. Patients with low radiomics signature value (risk score) had better RFS. The mean RFS at 1-, 2- and 3-year showed significant difference between the both subgroups in the training dataset (*P* = 0.004, *P* < 0.001, *P* = 0.024), but in the validation dataset, there was significant difference between the two groups (*P* = 0.044) only for 3-year, for 1- and 2-year no significant difference (*P* = 0.2169, *P* = 0.3402).

6. Nomogram construction and evaluation

**Table 3:** Predictive performance for RFS of the proposed models

Models	Training dataset *N* = 129	Validation dataset *N* = 55
	C-index (95%CI)	C-index (95%CI)
Clinical model		
clinicopathologic feature	0.649 (0.592–0.706)	0.556 (0.471–0.641)
Radiomics model		
Arterial phase	0.767 (0.702–0.832)	0.694 (0.623–0.832)
Portal vein phase	0.757 (0.692–0.821)	0.736 (0.632–0.841)
Parenchymal phase	0.789 (0.723–0.853)	0.686 (0.582–0.791)
All phases	0.791 (0.726–0.856)	0.690 (0.586–0.795)
Combined model		
Arterial phase + Clinicopathologic feature	0.797 (0.732–0.862)	0.732 (0.628–0.837)
Portal vein phase + Clinicopathologic feature	0.792 (0.727–0.857)	0.755 (0.651–0.860)
Parenchymal phase + Clinicopathologic feature	0.806 (0.741–0.871)	0.728 (0.624–0.834)
All phases + Clinicopathologic feature	0.809 (0.744–0.874)	0.724 (0.620–0.829)

C-index (Harrell concordance index) indicates the predictive performance

**Table 4:** RFS and recurrence rates in the high-risk and low-risk groups

	Training Dataset *N* = 129			Validation Dataset N = 55		
	High-Risk Group	Low-Risk Group	*P*-value	High-Risk Group	Low-Risk Group	*P*-value
No. of patients (%)	65 (50.4)	64 (49.6)		24 (43.6)	31 (56.4)	
1-year RFS Median, (IQR)	4.5 (2.0–6.25)	7 (5–10)	0.004	4 (2–5)	8 (2–8)	0.2169
2-year RFS Median, (IQR)	5 (2.25–9)	11 (7–16)	< 0.001	6.5 (3–15)	10 (5.75–15.5)	0.3402
3-year RFS Median, (IQR)	6 (3.0–16.0)	13 (7.5–19)	0.024	9 (3.5–16.5)	17.5 (8.0–26)	0.044
No. of recurrences (%)						
At 1 year	44 (34.1)	16 (12.4)		13 (23.6)	9 (14.5)	
At 2 year	54 (41.9)	29 (22.5)		20 (36.4)	14 (20.0)	
At 3 year	63 (48.8)	39 (30.2)		23 (41.8)	22 (40.0)	

**Fig. 4:**
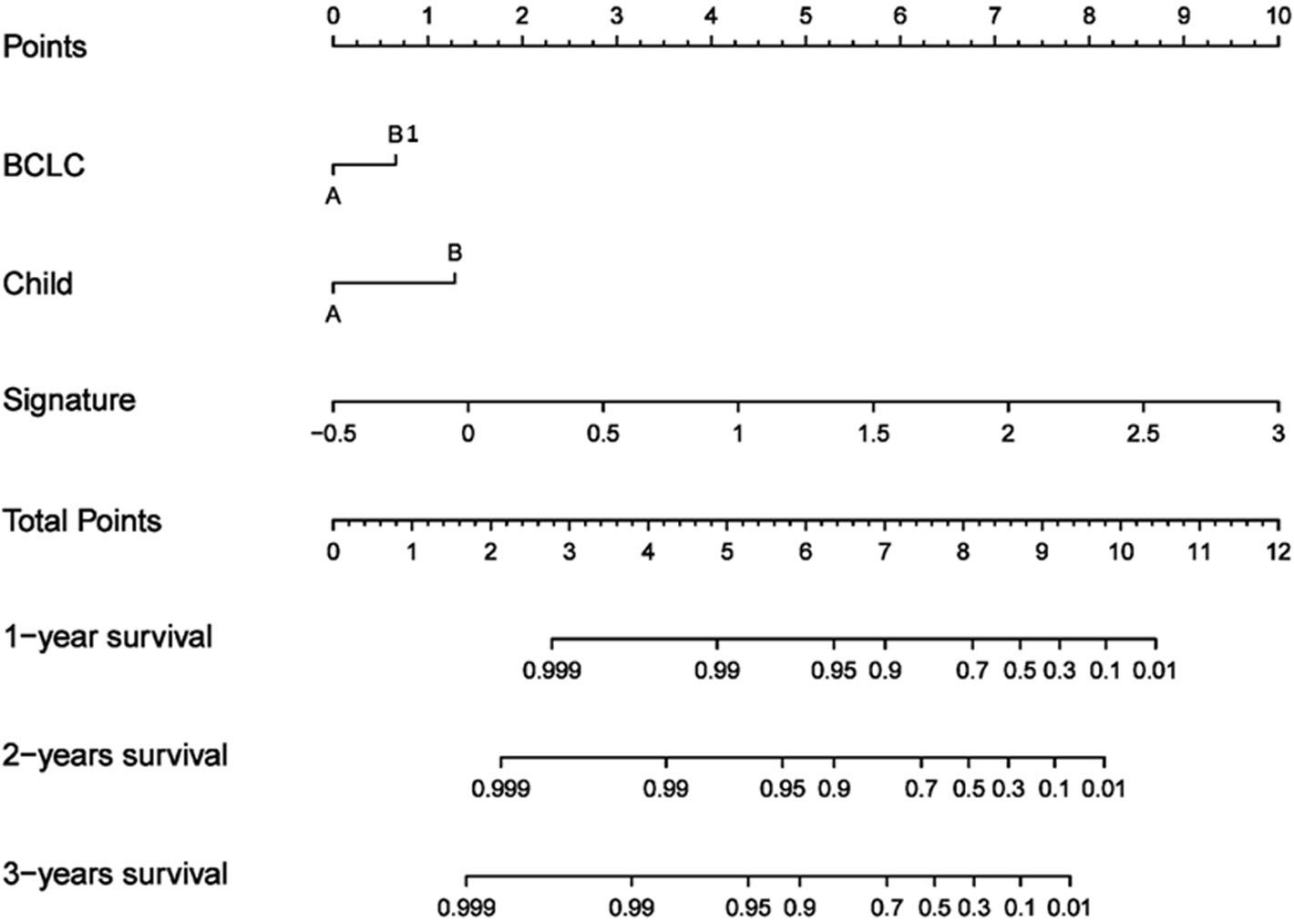
The nomogram may have the potential to individually predict RFS in a particular patient after curative ablation accordingto his clinicopathologic feature and radiomics signature. To use the nomogram, locate the margin according to the patient information, draw a line straight up to the points axis to obtain the score associated with BCLC. Repeat for the Child-Pugh and radiomics signature separately. The final score was obtained by summing all the single scores. Locate it on the total points axis and draw a line straight down to the bottom axis, the estimated survival probability could be determined

**Fig. 5:**
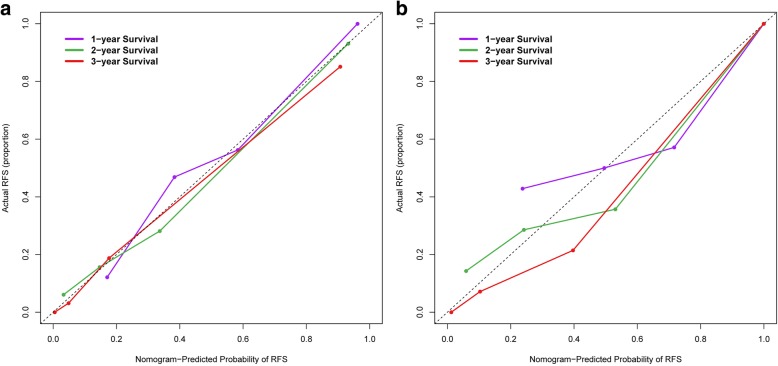
Calibration curves of the combined nomogram in the (**a**) training and (**b**) validation datasets. The y-axis represents the actual recurrence-free survival (RFS). The x-axis represents the predicted RFS possibility. The diagonal dashed line indicates the ideal prediction by a perfect model

As the combined model incorporating the portal venous phase radiomics signature and the clinicopathological factor had the best predictive performance, we built a nomogram based on this final model (Fig. 4). Furthermore, calibration curves of the combined nomogram were plotted for the training and validation datasets (Fig. 5). The Hosmer-Leme show test of the model showed non-significant differences in the training (*P* = 0.791) and validation (*P* = 0.471) datasets, which demonstrated a satisfying agreement.

## Discussion

The use of radiomics in medicine is still in its infancy and additional studies are necessary to examine the radiomics signatures that could predict patient outcomes. To date, no study used radiomics to investigate the prediction of HCC recurrence after ablation. Therefore, this study aimed to develop and validate radiomics signatures that could predict RFS after curative ablation. A large number of radiomic features in our study were extracted, as it may cause overfitting compared with the number of cases, we performed feature reduction and selection for the final model building. Accordingly, the radiomics model constructed with optimal features subset achieved satisfying performance. Moreover, the results of nomogram indicated that this study successfully built a combined model that integrated clinicopathological factors and radiomics features. For BCLC stage 0-B2 HCC, ablation and surgery are among the preferred options. Especially, ablation can be conducted for patients who are not suitable for surgery [13]. These ablation techniques destroy tumor cells locally but the destroyed tumor is not removed from the body [37], which can release intact tumor proteins that may serve as some kind of vaccine against eventual HCC recurrence [38]. Therefore, the recurrence patterns and odds of ablations may be not the same as those of surgical resection. Studies that specifically examine the outcomes of ablation are therefore required.

The BCLC staging system has been endorsed and recommended by multiple authoritative academic organizations for prognosis and treatment stratification of HCC patients. Indeed, it includes patient-related factors, tumor extent and liver function, and an algorithmic component for management. The BCLC staging and Child-Pugh score are included in a number of models predicting HCC recurrence [38–40], they are also important factors associated RFS of HCC patients in our present study.

So far, one radiomics study of HCC examined the risk of ER after surgical resection. Indeed, Zhou et al. [25] developed a radiomics signature using 215 HCC of BCLC 0-C stage patients after hepatectomy to predict ER (<1 year in their study), but not of 2- and 3-year recurrence. Because patients with HCC of BCLC C stage are not suitable for curative ablation, it was not included in our study. A number of authors assessed the biological behavior of HCC using imaging features [18–20]. Similarly, radiomics signatures were built for recurrence prediction of a variety of cancers after surgery [25, 41–44], but it has to be noted that ablation is usually not indicated in those cancers. The present study suggests that the combination of the portal venous phase radiomics signature and the clinicopathological data provides fair to good results in a technically homogeneous dataset, but to really establish a model like this, external validation and larger dataset would be required.

The present study has a number of strengths. First, all patients were diagnosed HCC by percutaneous biopsy before treatment. In addition, CT-guided percutaneous curative ablation (i.e., complete response according to modified RECIST (mRECIST) standard) [45] was achieved in all patients, which is comparable to radical surgical resection [46]. Secondly, all patients were followed > 3 years, which can be considered mid-term follow-up. Finally, the radiomics approach uses CECT images, which are routinely used for the diagnosis and follow-up of patients with HCC and widely available.

Furthermore, it has to be highlighted that radiomics results from specialized software instead of traditional imaging using the naked eye. In addition, radiomics captures more information about the tumor than percutaneous biopsy or histopathological examination of the surgical specimen, with little additional cost and good predictive outcomes [19]. Radiomics is still in its infancy, but a number of studies show promising results in a variety of cancers [25, 41–44]. Therefore, tumor recurrence prediction could be improved using radiomics nomograms. The present study improves the radiomics approach by including clinicopathological features in the final model, which has hardly been done before. One previous study suggested the use of the immunohistochemical markers CK19 and GPC3 for the prediction of HCC recurrence [47]. Future models could use a combination of radiomics, clinicopathological factors, and immunohistochemical characteristics of the HCC. In addition, the radiomics models should be directly compared with the available and widely used clinical models of HCC prognosis [14–16]. This has to be examined in the future.

Of course, the present study is not without limitations. This was a retrospective single center study spanning a long period of time. Therefore, several biases related to the treatment method and imaging could be present. In addition, the sample size was relatively small, external validation and larger datasets are needed to validate and refine our results.

## Conclusions

The present study establishing a model for the prediction of HCC recurrence after curative ablation, that combined radiomics signature in the portal venous phase with clinicopathological features. This model could help stratify the patients in order to adopt the most appropriate follow-up and interventional strategy.

## Additional file

Additional file 1: **Table S1.** Selected features for CT image in different phases. **Table S2.** Detailed information of the selected features in radiomics models. **Table S3.** Radiomics signature for CT image in different phases. (DOC 57 kb)

## Abbreviations

AFP: alfa fetal protein
BCLC: Barcelona Clinic Liver Cancer
CECT: contrast enhanced computed tomography
CEMRI: contrast enhanced magnetic resonance imaging
Cryo-A: cryoablation
DICOM: digital imaging and communications in medicine
ECOG: Eastern Cooperative Oncology Group
HCC: hepatocellular Carcinoma
ICC: intra-class correlation coefficient
LASSO: least absolute shrinkage and selection operator
MRMRA: minimum redundancy maximum relevance algorithm
MWA: microwave ablation
PET-CT: Positron Emission Tomography-Computed Tomography
RFA: radiofrequency ablation
RFS: recurrence free survival
ROI: Regions of interest
TACE: transarterial chemoembolization
