# Supplementary material for: Prediction early recurrence of hepatocellular carcinoma eligible for curative ablation using a Radiomics nomogram
Source: Cancer Imaging. 2019 Apr 26;19:21. doi: 10.1186/s40644-019-0207-7 (PMC6485136; doi:10.1186/s40644-019-0207-7)
Supplement: Supplementary file 1 — Table S1. Selected features for CT image in different phases. Table S2. Detailed information of the selected features in radiomics models. Table S3. Radiomics signature for CT image in different phases. (DOC 57 kb) [file 40644_2019_207_MOESM1_ESM.doc]

**Supplementary Table 1.** Selected features for CT image in different phases

|  | Arterial phase  (N=5) | Portal vein phase  (N=5) | Parenchymal phase  (N=10) |
| --- | --- | --- | --- |
| Features | Coif3_glcm_maximum_probability | Coif3_glcm_entropy | Coif4_glszm_SZLGE |
|  | Coif1_glrlm_LRLGLE | Coif1_glszm_LZLGE | Coif1_glrlm_LGLRE |
|  | Coif3_fos_mean | Coif4_glszm_LGLZE | ori_Max3D |
|  | Coif7_glszm_LGLZE | Coif3_glcm_maximum_probability | Coif7_fos_mean |
|  | Coif3_glcm_cluster_shade | Ori_fos_maximum | Coif4_glcm_correlation |
|  |  |  | Coif1_glrlm_LRHGLE |
|  |  |  | Coif2_glcm_inverse_variance |
|  |  |  | Coif3_glszm_SZHGE |
|  |  |  | Coif5_glcm_covariance |
|  |  |  | Coif8_glszm_SZE |

Features were selected by lasso modeling via the leave-one-out cross-validation in the separate three CT phases.

**Supplementary Table 2.** Detailed information of the selected features in radiomics models

| **Phases** | **Feature name** | **Formula** | **Content** |
| --- | --- | --- | --- |
| Arterial | Coif3_glcm_maximum_probability | maximum_probability  = $\max\{C(i,j)\}$ | The maximum probability of the Gray-Level Co-Occurrence Matrix of CT image transformed by wavelet filter XLHL |
|  | Coif1_glrlm_LRLGLE | LRLGLE  = | Long Run Low Gray Level Emphasis of Gray-Level Run-Length matrix of the CT image transformed by wavelet filter XLLL |
|  | Coif3_fos_mean | Entropy  = the mean intensity value | Mean intensity value of the CT image transformed by wavelet filter XLHL |
|  | Coif7_glszm_LGLZE | LGLZE  =$\sum_{i=1}^{N_{\text{g}}} \sum_{j=1}^{N_{z}} \left[\frac{Z(i,j)}{i^{2}}\right]$ | Low Gray Level Zone Emphasis of Gray-level size zone matrix of the CT image transformed by wavelet filter XHHL |
|  | Coif3_glcm_cluster_shade | cluster_shade  =$\sum_{i=1}^{N_{\text{g}}} \sum_{j=1}^{N_{g}} [i+j-\mu_{x}(i)-\mu_{y}(j)]^{3}C(i,j)$ | Cluster shade of the Gray-Level Co-Occurrence Matrix of CT image transformed by wavelet filter XLHL |
| Portal vein | Coif3_glcm_entropy | Entropy  =$-\sum_{i=1}^{N_{\text{g}}} \sum_{j=1}^{N_{g}} C(i,j)\log_{2}[C(i,j)]$ | Entropy of the Gray-Level Co-Occurrence Matrix of CT image transformed by wavelet filter XLHL |
|  | Coif1_glszm_LZLGE | LZLGE  =$\sum_{i=1}^{N_{\text{g}}} \sum_{j=1}^{N_{r}} \left[\frac{Z(i,j)j^{2}}{i^{2}}\right]$ | Large Zone Low Gray-Level Emphasis of Gray-level size zone matrix of the CT image transformed by wavelet filter XLLL |
|  | Coif4_glszm_LGLZE | LGLZE  = $\sum_{i=1}^{N_{\text{g}}} \sum_{j=1}^{N_{z}} \left[\frac{Z(i,j)}{i^{2}}\right]$ | Low Gray Level Zone Emphasis of Gray-level size zone matrix of the CT image transformed by wavelet filter XLHH |
|  | Coif3_glcm_maximum_probability | maximum_probability  = $\max\{C(i,j)\}$ | Maximum probability of the Gray-Level Co-Occurrence Matrix of CT image transformed by wavelet filter XLHL |
|  | Ori_fos_maximum | fos_maximum  =$The\text{ }maximum\text{ }intensity\text{ }value\text{ }of\text{ X}$ | Maximum intensity value of the original image |
| Parenchymal | Coif4_glszm_SZLGE | SZLGE  =$\sum_{i=1}^{N_{\text{g}}} \sum_{j=1}^{N_{z}} \left[\frac{Z(i,j)}{i^{2}j^{2}}\right]$ | Small Zone Low Gray Level Emphasis of Gray-level size zone matrix of the CT image transformed by wavelet filter **XLHH** |
|  | Coif1_glrlm_LGLRE | LGLRE  = | Low Gray Level Run Emphasis of Gray-Level Run-Length matrix of the CT image transformed by wavelet filter **XLLL** |
|  | ori_Max3D | Max3D  = maximum three-dimensional tumor diameter | Maximum three-dimensional tumor diameter of the original CT image |
|  | Coif7_fos_mean | fos_mean  =$\frac{1}{N}\sum_{i=1}^{N_{\text{g}}} X(i)$ | Mean intensity of grey histogram of CT image transformed by wavelet filter XHHL |
|  | Coif4_glcm_correlation | Correlation  =$\sum_{i=1}^{N_{\text{g}}} \sum_{j=1}^{N_{g}} ijC(i,j)$ | Correlation of the Gray-Level Co-Occurrence Matrix of CT image transformed by wavelet filter XLHL |
|  | Coif1_glrlm_LRHGLE | LRHGLE  = | Long Run High Gray Level Emphasis of Gray-Level Run-Length matrix of the CT image transformed by wavelet filter XLLL |
|  | Coif2_glcm_inverse_variance | inverse_variance  =$\sum_{i=1}^{N_{\text{g}}} \sum_{j=1}^{N_{g}} \frac{C(i,j)}{\text{1+(}\frac{\left\vert i-j\right\vert^{2}}{N^{2}}\text{)}}$ | Inverse variance of the Gray-Level Co-Occurrence Matrix of CT image transformed by wavelet filter XLHL |
|  | Coif3_glszm_SZHGE | SZHGE  =$\sum_{i=1}^{N_{\text{g}}} \sum_{j=1}^{N_{z}} \left[\frac{Z(i,j)i^{2}}{j^{2}}\right]$ | Small Zone High Gray-Level Emphasis of Gray-level size zone matrix of the CT image transformed by wavelet filter XLHL |
|  | Coif5_glcm_covariance | Covariance  =$\sum_{i=2}^{2N_{\text{g}}} (i-SE)^{2}P_{x+y}(i)$ | Sum of covariance of the Gray-Level Co-Occurrence Matrix of CT image transformed by wavelet filter XLHL |
|  | Coif8_glszm_SZE | SZE  =$\sum_{i=1}^{N_{\text{g}}} \sum_{j=1}^{N_{z}} \left[\frac{Z(i,j)i^{2}}{j^{2}}\right]$ | Small Zone Emphasis of Gray-level size zone matrix of the CT image transformed by wavelet filter XLHH |

X is the intensity value of the original image.

XLLL, XLLH, XLHL, XLHH, XHLL, XHLH, XHHL, and XHHH are the intensity value of the transformation images from the original image by eight three-dimensional wavelet filters. L: low-pass filter; H: low-pass filter. For example, XLHL represent the intensity value resulting from directional filtering of X with a low-pass filter along the x-direction, a high pass filter along the y-direction and a low-pass filter along the z-direction.


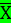

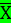
 is median intensity value of X.


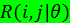

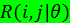
 is the value of row i and column j in the Gray-Level Run-Length Matrix for a direction
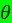

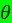
.


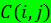

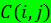
 is the value of row i and column j in the Gray-Level Co-Occurrence Matrix.


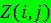

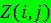
 is the value of row i and column j in the Gray-Level Size Zone Matrix.


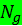

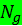
 is the number of discrete intensity values in the image.


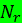

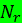
 is the number of different run lengths.


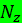

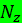
 is the size of the largest homogeneous region .


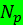

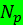
 is the number of voxels in the image .


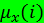

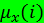
 is the mean of row i.


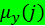

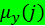
 is the mean of column j.


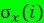

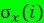
 is the standard deviation of row i.


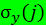

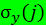
 is the standard deviation of column j.

**Supplementary Table 3.** Radiomics signature for CT image in different phases

| **Phase** | **Radiomics signature** |
| --- | --- |
| Arterial | Signature = 2.750826249-  0.022446033*N25_Coif3_glcm_maximum_probability-  0.054947769* N25_Coif1_glrlm_LRLGLE +  0.008390395* N25_Coif3_fos_mean +  .152563563*N25_Coif7_glszm_LGLZE-0.034839997*N25_Coif3_glcm_cluster_shade |
| Portal venous | Signature = 2.633335158+0.062617223*N25_Coif3_glcm_entropy -  0.025120597*N25_Coif1_glszm_LZLGE+  0.034852387*N25_Coif4_glszm_LGLZE-  0.061244532* N25_Coif3_glcm_maximum_probability +  0.055584297* N25_ori_fos_maximum |
| Parenchymal | Signature = 3.410207937+0.207406911*N25_Coif4_glszm_SZLGE–  0.082837573*N25_Coif1_glrlm_LGLRE+  0.060390091*N25_ori_Max3D+  0.035731692*N25_Coif7_fos_mean-  0.124767859* N25_Coif4_glcm_correlation+  0.047483351*N25_Coif1_glrlm_LRHGLE+  0.00022*N25_Coif2_glcm_inverse_variance+ 0.067083626*N25_Coif3_glszm_SZHGE+  0.071125387*N25_Coif5_glcm_covariance- 0.045437444*N25_Coif8_glszm_SZE |
